# Predictive value of peri-chemotherapy hematological parameters for febrile neutropenia in patients with cancer

**DOI:** 10.3389/fonc.2024.1380195

**Published:** 2024-08-19

**Authors:** Hongyuan Jia, Long Liang, Xue Chen, Wenzhong Zha, Wei Diao, Wei Zhang

**Affiliations:** ^1^ Department of Radiation Oncology, Sichuan Clinical Research Center for Cancer, Sichuan Cancer Hospital and Institute, Sichuan Cancer Center, Affiliated Cancer Hospital of University of Electronic Science and Technology of China, Chengdu, China; ^2^ Cancer Center, Sichuan Taikang Hospital, Chengdu, Sichuan, China; ^3^ Department of Biomedical Informatics. Sichuan Cancer Hospital and Institute, Sichuan Cancer Center, School of Medicine, University of Electronic Science and Technology of China, Chengdu, China; ^4^ Department of Nuclear Medicine, Sichuan Clinical Research Center for Cancer, Sichuan Cancer Hospital and Institute, Sichuan Cancer Center, Affiliated Cancer Hospital of University of Electronic Science and Technology of China, Chengdu, China

**Keywords:** febrile neutropenia, chemotherapy, hematological parameters, prediction, nomogram

## Abstract

**Objective:**

The aim of this study was to compare hematological parameters pre- and early post-chemotherapy, and evaluate their values for predicting febrile neutropenia (FN).

**Methods:**

Patients diagnosed with malignant solid tumors receiving chemotherapy were included. Blood cell counts peri-chemotherapy and clinical information were retrieved from the hospital information system. We used the least absolute shrinkage and selection operator (LASSO) method for variable selection and fitted selected variables to a logistic model. We assessed the performance of the prediction model by the area under the ROC curve.

**Results:**

The study population consisted of 4,130 patients with common solid tumors receiving a three-week chemotherapy regimen in Sichuan Cancer Hospital from February 2019 to March 2022. In the FN group, change percentage of neutrophil count decreased less (−0.02, CI: −0.88 to 3.48 vs. *−*0.04, CI: −0.83 to 2.24). Among hematological parameters, lower post-chemotherapy lymphocyte count (OR 0.942, CI: 0.934–0.949), change percentage of platelet (OR 0.965, CI: 0.955–0.975) and higher change percentage of post-chemotherapy neutrophil count (OR 1.015, CI: 1.011–1.018), and pre-chemotherapy NLR (OR 1.002, CI: 1.002–1.002) predicted an increased risk of FN. These factors improved the predicting model based on clinical factors alone. The AUC of the combination model was 0.8275.

**Conclusion:**

Peri-chemotherapy hematological markers improve the prediction of FN.

## 1 Introduction

Chemotherapy-induced febrile neutropenia (FN) is one of the most concerning sequelae in patients with cancer undergoing chemotherapy (1). It often leads to serious infections and causes dose reduction and delays that may impair survival outcomes (2). Current guidelines recommend prophylactic granulocyte colony-stimulating factor (G-CSF) based on the estimated FN risk (3). They are mainly based on a chemotherapy regimen, considering specific individual patient characteristics. However, until now, there is no widely accepted mechanism to quantify patient-specific risk.

Several models that focused on certain predictors have been proposed. These factors include tumor type, number of chemotherapy cycles, and chemotherapy regimen (4). Individual patient characteristics, such as age and comorbidities, are also associated with FN risk (5). Lyman’s model is a widely used risk model published in 2011 with an area under the receiver operating characteristics curve (AUC) of 0.81. Its risk factors included patient-specific variables such as age, receipt of prior chemotherapy, cancer type, white blood cell count, and liver and renal function parameters before chemotherapy (6). In a recent external validation study, the Lyman model demonstrated moderate predicting value with an AUC of 0.7475. It also included too many variables, which limits an easy use in clinical practice.

Blood cell counts are essential and economic tests during chemotherapy. They are needed before chemotherapy to determine whether chemotherapy is feasible. Blood cell counts before chemotherapy such as white blood cell count, absolute neutrophil count, and lymphocyte count have been shown of certain value in predicting FN (7, 8). Blood cell counts are also commonly performed within a few days of chemotherapy in clinical practice, while severe neutropenia usually occurs 7 to 10 days after chemotherapy. Except for hematological parameters sampled before chemotherapy, these parameters early after chemotherapy may function better to predict FN risk. Moreover, hematological parameter-derived indexes such as systemic inflammatory index (SII), neutrophil–lymphocyte ratio (NLR), and platelet–lymphocyte ratio (PLR) were related to host immune status. They are widely used in predicting the prognosis of cancer as well as some non-malignant diseases (9–11). However, it has not been fully elucidated whether blood cell counts before and early after chemotherapy and their derived indicators can increase the predictive value in addition to patient-specific variables.

In the current study, we compared pre- and early post-chemotherapy hematological parameters between non-FN and FN groups. We selected key factors for predicting FN from all variables by the least absolute shrinkage and selection operator (LASSO) method. We then establish three models based on hematological or clinical features alone or a combination of both and compare their performances. This study is of the largest data to date to compare pre- and early post-chemotherapy hematological parameters and analyze their predictive value for FN. We aimed to compare hematological parameters pre- and early post-chemotherapy, and evaluate their values for predicting FN.

## 2 Materials and methods

### 2.1 Study population

We retrospectively searched the hospital information system (HIS) for chemotherapy data between January 2019 and November 2022 at Sichuan Cancer Hospital. The center is a key center for cancer care in southwest China with 1,500 beds that provides services for over 100,000 patients per year. Inclusion criteria were patients with solid tumors receiving a three-week intravenous chemotherapy regimen, having blood cell counts 1 day before chemotherapy and early after chemotherapy (within 1 to 4 days), and at least three routine blood tests within 3 weeks after chemotherapy. Excluding criteria were incomplete or missing data on the hematological markers or clinical characteristics necessary for analysis. Chemotherapy regimens and dosage were chosen by treating medicines according to guidelines and individual patient conditions. The protocol was approved by the institutional review board and conducted in accordance with the Declaration of Helsinki and adhered to Good Clinical Practice guidelines. The requirement for written informed consent was waived by the institutional review board.

### 2.2 Study variables and outcome

Patients’ clinical features were automatically extracted using structured query language (SQL) from HIS, including tumor type, stage, age, sex, height, weight, concurrent radiation, prior surgery, comorbidities, cycles, and regimen of chemotherapy. We also searched the blood test database based on the list of patients and matched the treatment delivery date and blood test date. SII, NLR, and PLR were calculated. The changing rate (cr) and changing percentage (cp) were defined as the difference between pre- and post-chemotherapy values divided by the time interval or the pre-chemotherapy value; for example, the changing rate and change percentage of lymphocyte are defined as $crL=\left(postL-preL\right)/time\;interval$ and $cpL=\left(postL-preL\right)/preL$ . FN was defined as current or anticipated absolute neutrophil count less than 500/mm^3^ with a temperature of ≥101°F (38.3°C) or ≥100.4°F (≥38.0°C) sustained ≥1 h according to the definition of the Infectious Diseases Society of America.

### 2.3 Features selection and modeling

The data were divided into a training set and a validation set in a 7:3 ratio. Multicategory variables, such as cancer type and chemotherapy regimen, were encoded using one hot encoder. We used the LASSO method to select key factors and fitted them to logistic regression models based on clinical or hematological features, or a combination of both. Performances of the predicting models were evaluated by receiver operating characteristic curve (ROC) on the testing datasets. To help physicians to easily determine the risk of the disease developing after chemotherapy, a nomogram was developed using risk factors selected from the final multivariable regression model.

### 2.4 Statistics

Comparisons for continuous variables were performed using the Mann–Whitney test, and for categorical variables, the chi‐squared test or Fisher’s exact test was used. The level of significance was set at *p* < 0.05. All statistical analyses were carried out using the R software version 3.4.3 (https://www.R‐project.org/). For LASSO regression, R package glmnet (version 2.0–16) was used (12).

## 3 Results

### 3.1 Patient characteristics

Of the 5,440 patients who met the inclusion criteria, 1,310 were excluded from analysis for the following reasons: insufficient number of blood tests, incomplete information, and not following the 3-week intravenous chemotherapy regimen. The final dataset included 4,130 patients of 6,595 chemotherapy cycles. Among these, FN occurred in 623 (9.45%) of all cycles. The median cycle of chemotherapy was 2 (1–8). The median age was 54.00 (36.00–71.00) years or older. Among these 6,595 chemotherapy cycles, patients in 3,460 cycles (52.46%) were women. Cervical cancer (24.7%) was the most common diagnosis, followed by colorectal cancer (22.1%). The characteristics of the patients prior to each chemotherapy cycle are summarized in **Table 1**. The patients who had FN were typically women (*p* < 0.001), lower weight (*p* < 0.001), with concurrent radiation (*p* < 0.0010), and receiving specific chemotherapy drug such as docetaxel (*p* < 0.001).

**Table 1:** Patient characteristics.

Variable	Category	Non-FN	FN	All	*p*-value
**Age**	–	54.00, CI:36.00-71.00	55.00, CI:39.00-71.00	54.00, CI:36.00-71.00	0.0316
**BMI**	–	22.31, CI:17.90-28.08	22.03, CI:17.89-27.36	22.31, CI:17.90-28.04	0.0054
**BSA**	–	1.59, CI:1.37-1.85	1.55, CI:1.34-1.78	1.58, CI:1.37-1.85	0.0000
**Cycle of CH**	–	2.00, CI:1.00-8.00	2.00, CI:1.00-6.00	2.00, CI:1.00-8.00	0.0000
**Height**	–	160.00, CI:150.00-173.00	158.00, CI:149.70-170.00	160.00, CI:150.00-173.00	0.0000
**Weight**	–	58.00, CI:45.00-75.00	56.00, CI:43.00-70.00	58.00, CI:45.00-75.00	0.0000
**Cancer type**	Breast	78(1.31%)	25(4.01%)	103(1.56%)	0.0000
	Cervix	1409(23.59%)	222(35.63%)	1631(24.73%)	
	Colorectum	1296(21.70%)	22(3.53%)	1318(19.98%)	
	Endometrium	182(3.05%)	38(6.10%)	220(3.34%)	
	Esophagus	525(8.79%)	92(14.77%)	617(9.36%)	
	Head and Neck	384(6.43%)	45(7.22%)	429(6.50%)	
	Liver	280(4.69%)	5(0.80%)	285(4.32%)	
	Lung	792(13.26%)	67(10.75%)	859(13.03%)	
	NPC	613(10.26%)	70(11.24%)	683(10.36%)	
	Ovum	243(4.07%)	27(4.33%)	270(4.09%)	
	Stomach	170(2.85%)	10(1.61%)	180(2.73%)	
**Diabetes**	No	5773(96.67%)	610(97.91%)	6383(96.79%)	0.0794
	Yes	199(3.33%)	13(2.09%)	212(3.21%)	
**Hypertension**	No	5620(94.11%)	596(95.67%)	6216(94.25%)	0.1059
	Yes	352(5.89%)	27(4.33%)	379(5.75%)	
**Metastasis**	No	5511(92.28%)	578(92.78%)	6089(92.33%)	0.0001
	Yes	461(7.72%)	45(7.22%)	506(7.67%)	
**Radiation**	No	3855(64.55%)	349(56.02%)	4204(63.75%)	0.0000
	Yes	2117(35.45%)	274(43.98%)	2391(36.25%)	
**Sex**	Female	3056(51.17%)	404(64.85%)	3460(52.46%)	0.0000
	Male	2916(48.83%)	219(35.15%)	3135(47.54%)	
**Prior surgery**	No	3908(65.44%)	442(70.95%)	4350(65.96%)	0.0073
	Yes	2064(34.56%)	181(29.05%)	2245(34.04%)	
**Regime**	Carboplatin	1180(11.36%)	165(14.60%)	1345(11.67%)	0.0000
	Cisplatin	2014(19.38%)	242(21.42%)	2256(19.58%)	
	Docetaxel	684(6.58%)	251(22.21%)	935(8.12%)	
	Etoposide	176(1.69%)	19(1.68%)	195(1.69%)	
	Fluorouracil	1495(14.39%)	39(3.45%)	1534(13.31%)	
	Ifosfamide	21(0.20%)	2(0.18%)	23(0.20%)	
	Irinotecan	348(3.35%)	15(1.33%)	363(3.15%)	
	Lobaplatin	281(2.70%)	45(3.98%)	326(2.83%)	
	Nedaplatin	521(5.01%)	68(6.02%)	589(5.11%)	
	Oxaliplatin	1350(12.99%)	28(2.48%)	1378(11.96%)	
	Paclitaxel	2062(19.84%)	248(21.95%)	2310(20.05%)	
	Pemetrexed	259(2.49%)	8(0.71%)	267(2.32%)	
**RDI**	–	0.914(CI:0.6397-1.046)	0.948(CI:0.569-1.051)	0.9185(CI:6265-1.0471)	0.0035

BMI, body mass index; BSA, body surface area; CH, chemotherapy; CI, confidence interval; NPC, nasopharyngeal carcinoma; RDI, relative drug intensity; -, not applicable.

### 3.2 Comparison of hematological parameters

**Figure 1:**
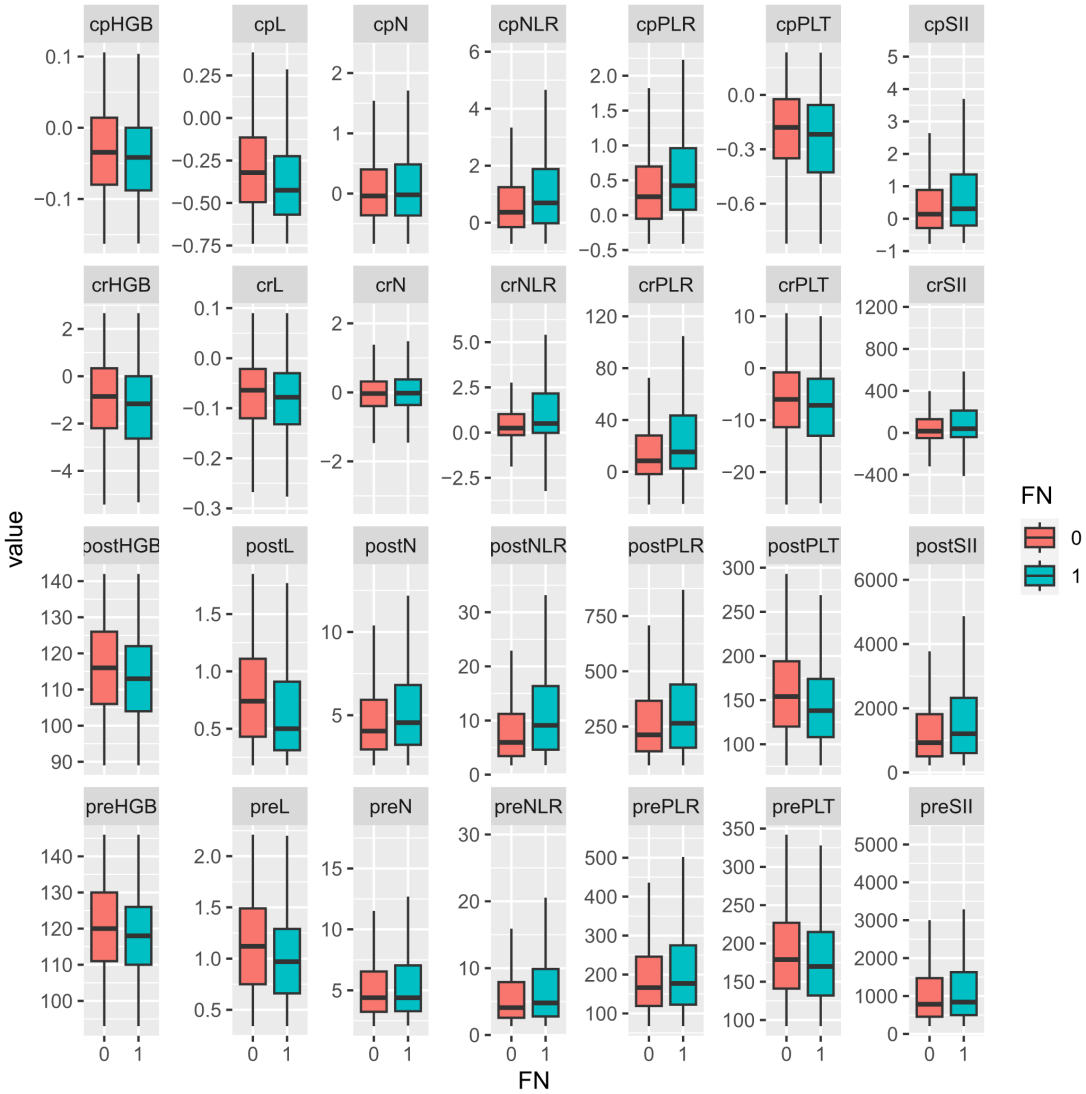
Comparison of hematological parameters and their changes between non-FN and FN groups. “0” for the non-FN group and “1” for the FN group. Prefix; cp, change percentage; cr, change rate; post, post-chemotherapy; pre, pre-chemotherapy. HGB, hemoglobulin; L, lymphocyte; N, neutrophil; NLR, neutrophil-to-lymphocyte ratio; PLR, platelet-to-lymphocyte ratio; PLT, platelet; SII, systemic immune inflammation index.

Median values for neutrophils, lymphocytes, platelets, hemoglobin, and their change rate and change percentage before and early after chemotherapy are presented in **Table 2**; **Figure 1**. SII, NLR, PLR, and their change rate were also calculated. Compared with the non-FN group, the FN group had lower early post-chemotherapy lymphocyte, platelet, and hemoglobin counts (0.46, CI: 0.13–1.58 vs. 0.74, CI: 0.18–1.88, 131.00, CI: 64.00–248.00 vs. 154.00, CI: 78.00–298.00, 112.00, CI: 86.00–137.00 vs. 116.00, CI: 88.00–143.00, respectively), while post-chemotherapy neutrophils were higher (4.48, CI: 1.23–14.89 vs. 4.05, CI: 2.02–13.30, respectively). Lymphocytes (−0.44, CI: −0.79 to 0.25 vs. −0.32, CI: −0.73 to 0.39) and platelets (−0.24, CI: −1.01 to 0.20 vs. −0.18, CI: −0.79 to 0.24) had deeper decreasing amplitude in the FN group; in addition, their decreasing rates were faster (−0.08, CI: −0.28 to 0.04 vs. −0.06, CI: −0.29 to 0.10 and −7.33, CI: −25.65 to 6.64 vs. −0.06, CI:−0.29 to 0.10). The FN group had higher SII, NLR, and PLR before and early after chemotherapy, and the increasing rate of these indicators after chemotherapy was also higher.

**Table 2:** Comparison of hematological parameters between non-FN and FN groups.

Variable	Non-FN	FN	Unit	*p*-value
**cpHGB**	−0.03, CI: −0.16 to 0.11	−0.05, CI: −0.19 to 0.10	–	0.0001
**cpL**	−0.32, CI: −0.73 to 0.39	−0.44, CI: −0.79 to 0.25	–	0.0000
**cpN**	−0.04, CI: −0.83 to 2.24	−0.02, CI: −0.88 to 3.48	–	0.3737
**cpNLR**	0.36, CI: −0.74 to 5.60	0.74, CI: −0.79 to 8.60	–	0.0000
**cpPLR**	0.26, CI: −0.41 to 2.28	0.43, CI: −0.41 to 2.69	–	0.0000
**cpPLT**	−0.18, CI: −0.79 to 0.24	−0.24, CI: −1.01 to 0.20	–	0.0000
**cpSII**	0.14, CI: −0.77 to 4.88	0.32, CI: −0.85 to 6.63	–	0.0005
**crHGB**	−0.86, CI: −5.40 to 2.75	−1.20, CI: −5.97 to 2.43	g/L*d	0.0002
**crL**	−0.06, CI: −0.29 to 0.10	−0.08, CI: −0.28 to 0.04	10^9^/L*d	0.0001
**crN**	−0.03, CI: −3.17 to 2.23	−0.02, CI: −3.88 to 2.82	10^9^/L*d	0.2462
**crNLR**	0.25, CI: −3.87 to 6.02	0.57, CI: −5.52 to 11.76	–	0.0000
**crPLR**	8.47, CI: −25.11 to 120.10	15.76, CI: −27.90 to 149.16	–	0.0000
**crPLT**	−6.00, CI: −26.33 to 10.81	−7.33, CI: −25.65 to 6.64	10^9^/L*d	0.0003
**crSII**	16.15, CI: −669.94 to 1,062.95	42.17, CI: −823.06 to 1,528.80	10^9^/L*d	0.0001
**postHGB**	116.00, CI: 88.00 to 143.00	112.00, CI: 86.00 to 137.00	g/L	0.0000
**postL**	0.74, CI: 0.18 to 1.88	0.46, CI: 0.13 to 1.58	10^9^/L	0.0000
**postN**	4.05, CI: 2.02 to 13.30	4.48, CI: 1.23 to 14.89	10^9^/L	0.0018
**postNLR**	5.88, CI: 1.69 to 34.92	9.96, CI: 1.86 to 52.48	–	0.0000
**postPLR**	211.60, CI: 73.08 to 921.57	272.00, CI: 85.08 to 1,041.25	–	0.0000
**postPLT**	154.00, CI: 78.00 to 298.00	131.00, CI: 64.00 to 248.00	10^9^/L	0.0000
**postSII**	926.92, CI: 221.34 to 6,083.53	1,235.56, CI: 211.06 to 7,687.20	10^9^/L	0.0000
**preHGB**	120.00, CI: 92.00 to 148.00	118.00, CI: 92.10 to 142.90	g/L	0.0001
**preL**	1.13, CI: 0.34 to 2.23	0.93, CI: 0.29 to 2.08	10^9^/L	0.0000
**preN**	4.39, CI: 2.06 to 17.67	4.40, CI: 2.00 to 20.44	10^9^/L	0.3489
**preNLR**	4.08, CI: 1.30 to 29.64	5.03, CI: 1.42 to 38.72	–	0.0000
**prePLR**	166.19, CI: 67.17 to 555.00	179.61, CI: 68.06 to 582.63	–	0.0107
**prePLT**	179.00, CI: 92.00 to 347.00	166.00, CI: 85.00 to 294.00	10^9^/L	0.0000
**preSII**	783.18, CI: 204.36 to 5,165.35	860.85, CI: 211.48 to 5,837.99	10^9^/L	0.0193

Cp, change percentage; Cr, change rate; L, lymphocyte; N, neutrophil; PLT, platelet; HGB, hemoglobin; SII, systemic immune inflammatory index; NLR, neutrophil–lymphocyte ratio; PLR, platelet–lymphocyte ratio; -, dimensionless.

### 3.3 Features selection and modeling

**Figure 2:**
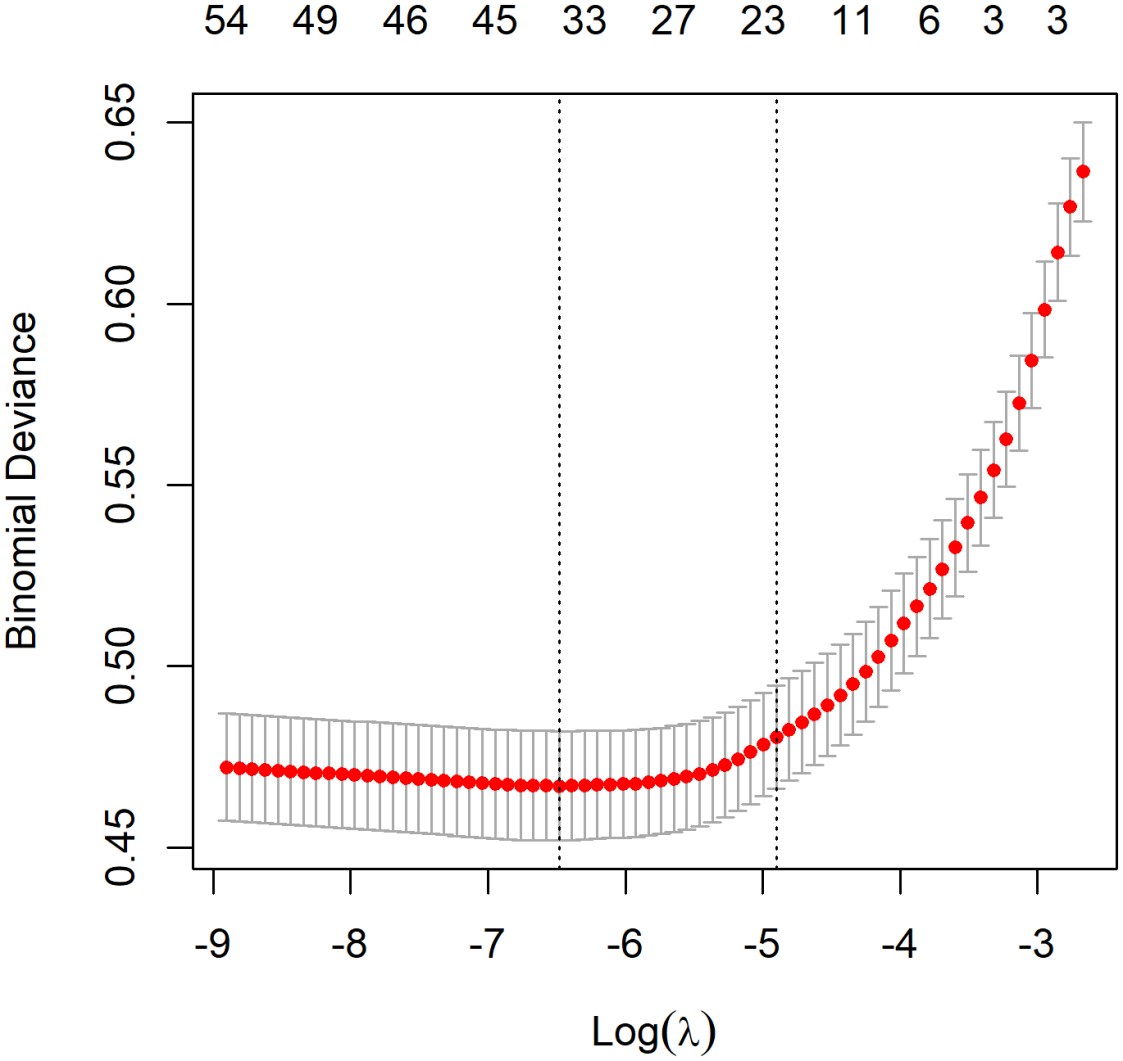
Feature selection by LASSO. A λ value of 0.0015289 was chosen, corresponding to the dotted line on the left.

**Figure 3:**
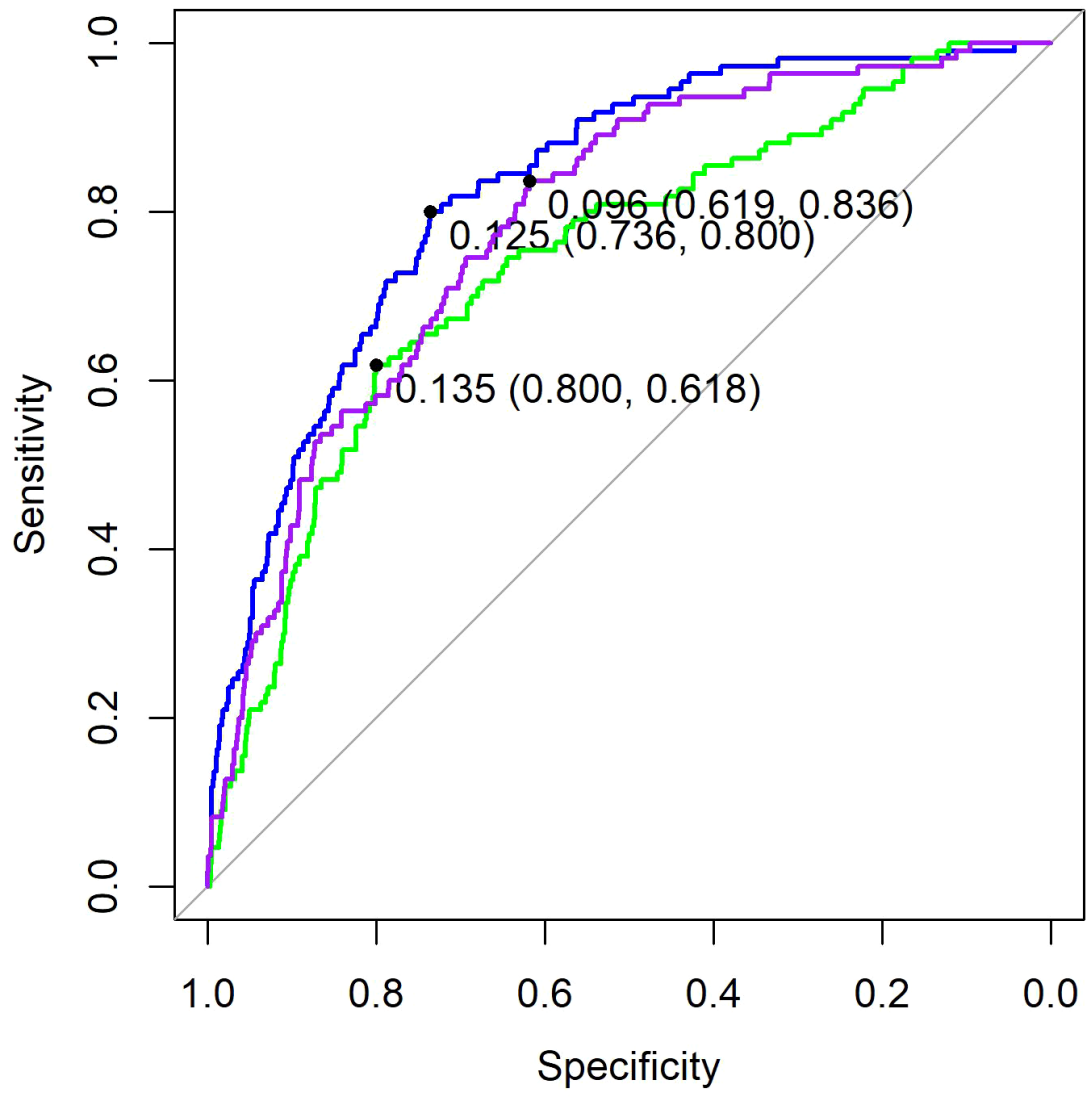
ROC of three models. Purple, the model based on clinical factors alone. Green, the model based on hematological factors alone. Blue, the model based on the combination of both.

**Figure 4:**
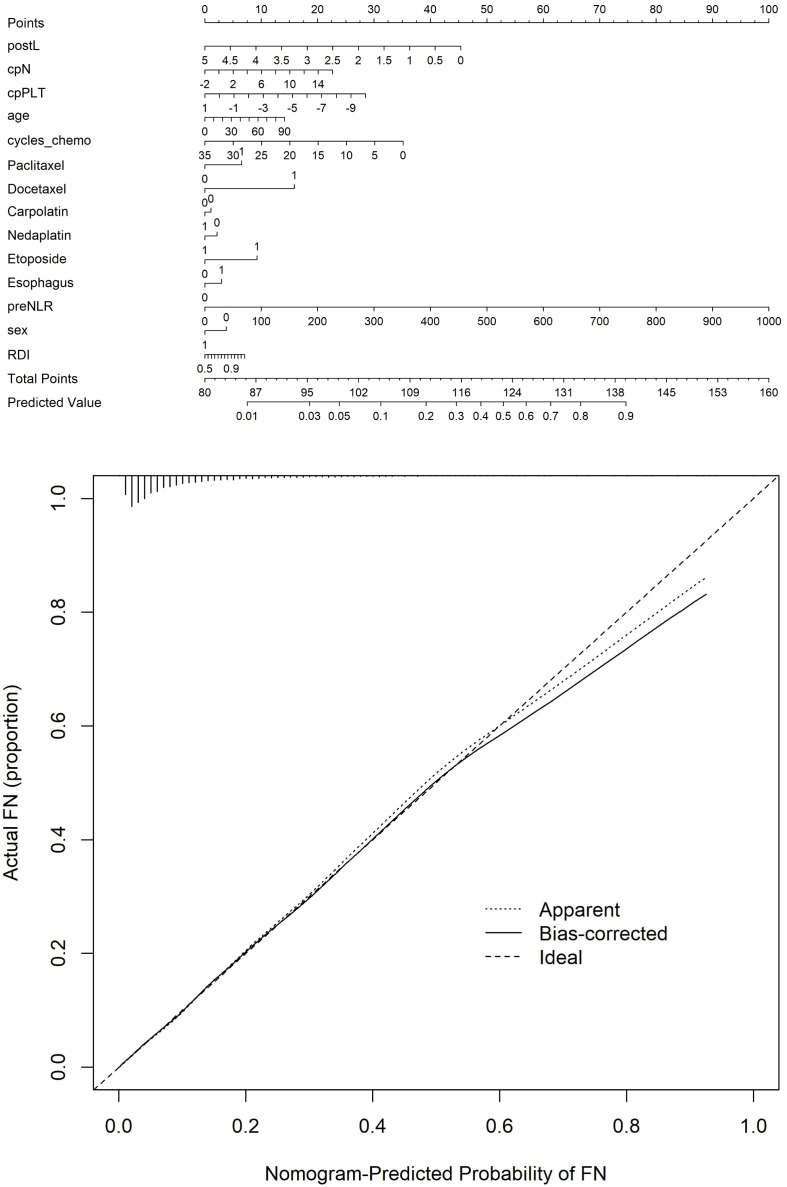
**(A)** Nomogram for predicting FN. **(B)** Calibration curve of the nomogram.

The optimal *λ* value of 0.0015289 was selected for the LASSO model by using 10-fold cross-validation (**Figure 2**). Using the LASSO method, 12 variables out of 28 hematological parameters were selected, including prePLT and preN. A total of 25 variables out of 37 clinical factors were selected, including Docetaxel and relative dose intensity (RDI). The selected hematological or clinical indicators are further fitted for a logistic regression model either alone or in combination. The combined model had the best prediction performance with an AUC of 0.8275, compared with 0.7412 and 0.7883, of models based on hematological or clinical parameters alone, respectively (**Figure 3**). Risk factors included in the combined model were postL; cpN; cpPLT; preNLR; age; sex; cycle of chemotherapy; specific drugs such as paclitaxel, docetaxel, carboplatin, nedaplatin, and etoposide; cancer of esophagus; and RDI. The odds ratios of the combined model are shown in **Table 3**. Among hematological parameters, lower postL (OR 0.942, CI: 0.934–0.949) and cpPLT (OR 0.965, CI: 0.955–0.975) and higher cpN (OR 1.015, CI: 1.011–1.018) and preNLR (OR 1.002, CI: 1.002–1.002) predicted an increased risk of FN. With a cutoff value of 0.125, the sensitivity and specificity were 0.800 and 0.736, respectively. The precision and accuracy of the model were 0.800 and 0.741, respectively. For the convenience of clinical utility, we constructed a nomogram based on the combination model (**Figure 4A**). Calibration curve indicated that the nomogram functions well (**Figure 4B**).

**Table 3:** OR of risk factor for models based on the combination of clinical and hematological parameters.

Risk factor	OR	*p*-value
**postL**	0.941, CI: 0.934–0.949	0.0000
**cpN**	1.015, CI: 1.012–1.018	0.0000
**cpPLT**	0.965, CI: 0.955–0.975	0.0006
**preNLR**	1.002, CI: 1.002–1.002	0.0000
**Age**	1.001, CI: 1.001–1.002	0.0002
**Cycle of CH**	0.995, CI: 0.993–0.996	0.0020
**Paclitaxel**	1.054, CI: 1.042–1.065	0.0000
**Carboplatin**	0.969, CI: 0.953–0.984	0.0454
**Docetaxel**	1.230, CI: 1.214–1.246	0.0000
**Nedaplatin**	0.950, CI: 0.933–0.968	0.0052
**Etoposide**	1.081, CI: 1.056–1.107	0.0008
**Esophagus**	1.038, CI: 1.023–1.052	0.0085
**Sex**	0.968, CI: 0.96–0.977	0.0003
**RDI**	1.124, CI: 1.089–1.16	0.0002

CI, confidence interval.

## 4 Discussion

Multiple previous studies have tried to predict FN using demographic, disease, and treatment characteristics. Clinical features such as older age, advanced disease, and early cycles of chemotherapy have all been reported to be associated with increased risk of severe neutropenia. However, it is unclear whether blood cell counts and their derivatives in the pre- and early post-chemotherapy period could further increase the predictive power for FN. In this study, we systematically compared them and found that platelets and lymphocytes decrease more in the FN group, while inflammatory index SII, NLR, and PLR increase more in FN group. Interestingly, a decreasing rate of neutrophil count was higher in the FN group. We then used the LASSO method to select key factors from a series of clinical and hematological factors. We further fitted three regression models based on selected hematological and clinical factors either alone or in combination. Compared with the prediction model based on clinical factors, the model combining clinical factors and hematological indicators has better predictive performance. In order to facilitate clinical application, we finally developed a nomogram for easy clinical utility.

The main tumor types in this study were cervical cancer and colorectal cancer, with breast cancer accounting for a smaller proportion. In previous studies, breast cancer usually occupies a larger proportion. Correspondingly, the proportion of patients receiving radiotherapy and with non-metastatic disease was relatively large in this study. We established a predictive model for FN in this new group for the first time.

In the non-FN and FN groups, as expected, cell counts of neutrophils, lymphocytes, platelets, and hemoglobulin levels decreased after chemotherapy. However, it is worth noting that in the FN group, the neutrophil count decreased less early after chemotherapy instead (−0.02, CI: −0.88 to 3.48 vs. −0.04, CI:−0.83 to 2.24). SII, NLR, and PLR, the indicators reflecting inflammation, all increased after chemotherapy, especially in the FN group. This is similar to the results of Cho et al. In their study, neutrophil counts were 3.329 ± 2.278 and 3.067 ± 1.343 5 days after chemotherapy in the FN and non-FN groups, respectively (*p* = 0.052), while there was no difference in the number of neutrophils between the two groups before chemotherapy (3.725 ± 1.550 vs. 3.815 ± 1.420, *p* = 0.383) (13). Therefore, we speculate that the lower decrease in neutrophil count after chemotherapy in the FN group is a manifestation of the potential inflammatory response superimposed on the direct neutrophil killing effect of chemotherapy drugs. Furthermore, elevated inflammatory markers may be caused by tumor necrosis. There may be a correlation between tumor sensitivity to drugs and bone marrow sensitivity to drugs. After the death of neutrophils mobilized into peripheral blood, they are not replenished by bone marrow regeneration (14). Thus, it is unstable and associates with more severe neutropenia.

The LASSO method can select key factors from a large number of variables. In one study, the prediction results of the LASSO model exceeded those of the traditional Lyman model (15). In this study, because there are too many independent variables, it is not suitable to perform logistic regression directly. Therefore, we first use the LASSO method to screen all variables, and then build a logistic regression model. The variables finally included in the model are cpN, cpPLT, preNLR, age, sex, cycle of chemotherapy, paclitaxel, docetaxel, carboplatin, nedaplatin, etoposide, esophagus, and RDI. We found that decreased cell counts of lymphocyte after chemotherapy predicted an increased risk of FN. Our results are consistent with some previous studies. Early lymphocyte count has the value of predicting FN, and the predictive significance on day 5 is greater than that on day 1 (16). The predictive value of PLT was proposed in a small retrospective analysis of patients with prostate cancer and NSCLC (17, 18). Our research further confirmed it with a much larger population. Previous studies also showed that lymphocyte count and immature platelet fraction level could predict recovery in patients who had developed severe neutropenia (19, 20). This indicated that changes in platelets and lymphocytes are more sensitive than changes in neutrophils during FN. Similarly, during the process of neutrophil decline, these two indicators can also help determine its severity. In the Jenkins study, pre-chemotherapy absolute neutrophil count and lymphocyte count predicted the risk of FN (7). However, in our study, when analyzed together with post-chemotherapy indicators and their change, except for pre-chemotherapy NLR, other pre-chemotherapy indicators did not have independent predictive effects.

Several clinical factors have been shown to affect the development of neutropenia in previous studies. Our study also found similar clinical predictors. These factors include age, sex, specific cancer type, and chemotherapy agents. Among various chemotherapy drugs, we found that docetaxel-containing chemotherapy regimens were significantly associated with an increased risk of FN. Previous studies indicate that concurrent radiation and previous surgery are risk factors for FN, while they failed to be of significance in the current model. There may be confouding effect regarding radiation dose, volume, location of target, and time to previous surgery. Thus, these results should be explained carefully and needed further studies.

The advantage of our model is the incorporation of early hematological indicators, which increased the AUC to 0.8275. The AUC value of our model is higher than that from historical data. Previous studies have reported the predictive value of some single hematological indicators, and many of them have a small sample size. Our study was the first comprehensive research on multiple hematological indicators before and after chemotherapy.

This study has certain limitations. First, the inherent susceptibility of patients to FN is related to their genetic profile, which should be included in analysis. Second, this study is a single-center retrospective study and may have bias. In the future, it is necessary to incorporate more predictive factors and to conduct multicenter prospective studies to further optimize the predictive model for FN after chemotherapy.

## 5 Conclusions

In summary, our research demonstrated that early post-chemotherapy hematological markers can improve the prediction of FN. The combined model can help in the early identification of patients with high FN risk, thereby accordingly adopting preventive measures.

## Data Availability

The raw data supporting the conclusions of this article will be made available by the authors, without undue reservation.
